# Sex drives colonic mucin sialylation in wild mice

**DOI:** 10.1038/s41598-024-57249-x

**Published:** 2024-03-23

**Authors:** Alexander R. Bennett, Iris Mair, Andrew Muir, Hannah Smith, Larisa Logunova, Andrew Wolfenden, Jonathan Fenn, Ann E. Lowe, Janette E. Bradley, Kathryn J. Else, David J. Thornton

**Affiliations:** 1 School of Biological Sciences, Faculty of Biology, Medicine and Health, Lydia Becker Institute of Immunology and Inflammation, University of Manchester, Manchester, UK; 2 School of Life Sciences, University of Nottingham, Nottingham, UK

**Keywords:** Glycobiology, Molecular ecology, Reproductive biology

## Abstract

Mucin protein glycosylation is important in determining biological properties of mucus gels, which form protective barriers at mucosal surfaces of the body such as the intestine. Ecological factors including: age, sex, and diet can change mucus barrier properties by modulating mucin glycosylation. However, as our understanding stems from controlled laboratory studies in house mice, the combined influence of ecological factors on mucin glycosylation in real-world contexts remains limited. In this study, we used histological staining with ‘Alcian Blue, Periodic Acid, Schiff’s’ and ‘High-Iron diamine’ to assess the acidic nature of mucins stored within goblet cells of the intestine, in a wild mouse population (*Mus musculus*). Using statistical models, we identified sex as among the most influential ecological factors determining the acidity of intestinal mucin glycans in wild mice. Our data from wild mice and experiments using laboratory mice suggest estrogen signalling associates with an increase in the relative abundance of sialylated mucins. Thus, estrogen signalling may underpin sex differences observed in the colonic mucus of wild and laboratory mice. These findings highlight the significant influence of ecological parameters on mucosal barrier sites and the complementary role of wild populations in augmenting standard laboratory studies in the advancement of mucus biology.

## Introduction

Mucus provides front line defence of the mucosal surfaces of the body such as the gut, lungs, and the cervix against physical, chemical or infectious insult^1^. The key structural components of the mucus barrier are gel-forming mucins^2^. These glycoproteins are extremely large polymers (2–100 MDa in humans), which are extensively glycosylated (up to 80% glycan, by weight)^3^. Mucin glycans are predominantly O-linked, with chains constructed via the sequential incorporation of monosaccharide units by glycosyltransferase enzymes^4^. Diversity in glycan chains arises through alterations in the sequence and linkage of monosaccharide sugars added to the mucin polypeptide. Variations in the glycan species present on gel forming mucins have implications for the protective properties of the mucus gel, the interactions between the mucus gel and microbiota, and the signalling capacity of mucins to host cells^5^. Altered mucin glycosylation is associated with pathologies such as inflammatory bowel disease, parasite infection and allergy^6–9^. Given its association with disease, control of mucin glycosylation at the intestinal mucus barrier is an active area of study. For example, the incorporation of sialic acid into mucins alters the rheological properties of the mucus gel, and sulphation is associated with resistance to endoparasitic infection^7,10^.

Numerous parameters alter the profile of mucin glycans at the intestinal barrier, including: diet, sex, age, microbiome and infection^11–13^. However, these effects have largely been evidenced through laboratory experiments. While laboratory studies have been successful in identifying individual influences on mucin glycosylation and uncovering underlying mechanisms, they fail to capture the combined influence of multiple factors on mucin glycosylation patterns. Recent studies employing wild *Mus musculus* exemplify the power of an ecological approach in identifying novel immune phenotypes which stem from environmental variation^14–17^.

The objective of this study was to investigate the primary factors influencing colonic mucin glycosylation, specifically sialylation and sulphation, in wild mice and to validate findings in controlled laboratory studies. A histological examination of colonic tissues in a wild population of *Mus musculus* was carried out and statistical modelling including ecological parameters was performed. Sex was identified as the primary driver of colonic goblet cell mucin glycan acidity. This observation was validated in laboratory housed C57BL/6 mice, where a significant increase in sialomucin content was observed in females compared to males. Additionally, molecular analysis of estrogen receptors and glycosyltransferase expression in wild animals revealed strong associations between the expression of Esr1 and sialyltransferase expression. These data implicate systemic sex hormone signalling as an important driver of intestinal mucus barrier properties.

## Results

### Sex is associated with acidic mucin staining in the proximal colon of wild *Mus musculus*

**Figure 1:**
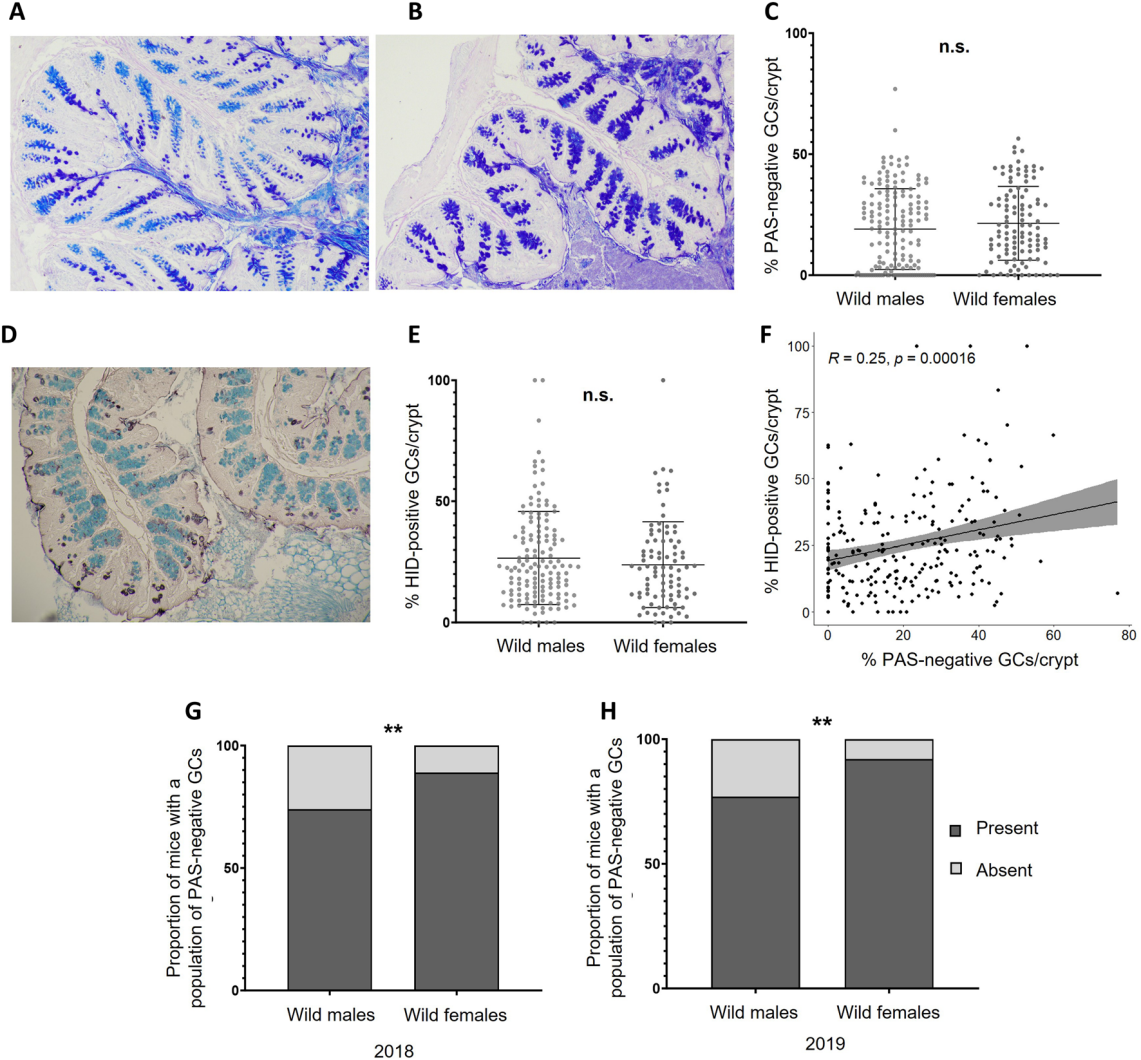
Sex is associated with acidic mucin staining in the proximal colon of wild *Mus musculus*. Representative images of ‘Alcian blue + Periodic acid and Schiff’s’ (AB + PAS) staining of proximal colon tissue, in wild mice with high proportions of PAS-negative staining (**A**) and no PAS-negative staining (**B**). The mean percentage of goblet cells (GCs) per crypt which stained PAS negative in IoM mice stratified by sex (**C**). A representative image of ‘high-iron diamine and Alcian blue’ (HID) staining of the proximal colon (**D**). The percentage of HID-positive cells per crypt amongst IoM mice stratified by sex (**E**). A correlation plot of the proportions of PAS negative goblet cells, and HID-positive GCs in the colons of all IoM mice (**F**). The proportions of mice with a presence or absence of PAS negative GCs, stratified by sex in 2018 (**G**) and 2019 (**H**). Each point represents an individual and is the average value of 10 crypts, n = 268 (157 males, 111 females). Error bars indicate the median ± SD. **p < 0.01, *n.s.* no significant difference. Significance determined by a chi-squared contingency analysis for categorical data, a Pearson test for correlative data and paired comparisons performed using a Mann–Whitney U test.

Following capture of animals and excision of proximal colonic tissue, Alcian blue combined with Periodic acid, Schiff’s (AB + PAS) staining was carried out. This allowed visualisation of the goblet cells, and examination of the acidic nature of mucins stored within secretory granules. Alcian blue is a positively charged dye molecule which binds to and stains negatively charged sialic acid and sulphate residues on mucin glycans. PAS stains neutral glycan structures, but can also stain non-acetylated sialic acids in mucin glycans (Table 1). Importantly for this study, AB-positive but PAS-negative staining goblet cells contain predominantly acidic glycan structures (Fig. 1A, Suppl. Fig. 1A,B).

**Table 1:** Chemical histological stains used in the analyses.

Stain	Alcian blue (AB)	Periodic acid Schiff’s (PAS)	Alcian blue + periodic acid and Schiff’s (AB + PAS)	High-iron diamine (HID)
*Substrate*	Binds to negatively charged sialic and sulphate residues	Binds vicinal diols on neutral glycan structures and non-acetylated sialic acid	Stains acidic and neutral glycans	Positively charged ammonium salts bind to negatively charged sulphate residues
Colour	Light blue	Magenta	Dark blue	Black

The proportion of PAS-negative staining goblet cells within the colons of 268 wild mice was highly variable, ranging from 0 to 77%, while 31.5% of mice (48 individuals) possessed no PAS-negative goblet cells (Fig. 1B,C, Suppl. Fig. 1C–E).

When comparing the cohort as a whole, the median percentage of goblet cells staining PAS-negative was similar between males, at 19.1% (inter quartile range (IQR) 34.4), and females, at 18.0% (IQR 22.5) (Fig. 1C). However, when the whole cohort was stratified by whether or not an individual possessed any PAS-negative goblet cells, a significantly greater proportion of female mice were seen to possess PAS-negative colonic goblet cells than male mice, a finding consistent across both 2018 (Fig. 1G) and 2019 (Fig. 1H). This suggests acidic glycan modifications are more widespread in the colonic goblet cells of wild female mice compared to male mice.

An example of staining along the entire colon, as well as a comparison between the proximal and distal regions is presented in Suppl. Fig. 2. Following these observations, model averaging using general linear models was undertaken to determine which environmental parameters were most associated with an animal’s possession of a population of PAS-negative goblet cells within the colon (parameters fully listed in Suppl. Fig. 3A). Sex was reported to be the most significant predictor of PAS-negative goblet cells in the wild mouse population (full model output in Suppl. Fig. 3B). Anti-Muc2 staining of tissue sections (Suppl. Fig. 4A–D) confirmed the presence of Muc2 in colonic goblet cells of the proximal colon.

To further understand the differences in mucin-glycosylation between male and female mice, High-Iron diamine (HID) staining was performed to determine if the variation in the percentage of PAS-negative goblet cells was due to alterations in the sulphate or sialic acid content of mucin glycans. The HID procedure allows the visualisation of sulphate structures specifically, and when counterstained with Alcian blue, indicates sulphomucin-containing goblet cells (staining black), and goblet cells containing exclusively sialomucins (light blue staining) (Fig. 1D, Suppl. Fig. 1F–H).

The percentage of HID-positive goblet cells/crypt varied between individual mice in the wild population, with the median percentage of HID-positive goblet cells per crypt being 22.6% (IQR 23.8) (Fig. 1E). The proportion of PAS-negative and HID-positive goblet cells correlated significantly, but with a weak coefficient (R = 0.25) (Fig. 1F). This suggests that although increased mucin sulphation may contribute to an increased prevalence of PAS-negative goblet cells, it may not be the major determinant. From this we conclude that increased sialylation of mucin glycans is the major change in glycosylation giving rise to increased population of PAS-negative staining goblet cells.

### In laboratory C57BL/6 mice, sulphation of colonic mucins is associated with the estrus cycle of female mice, but colonic mucin sialylation underpins the difference between males and females

**Figure 2:**
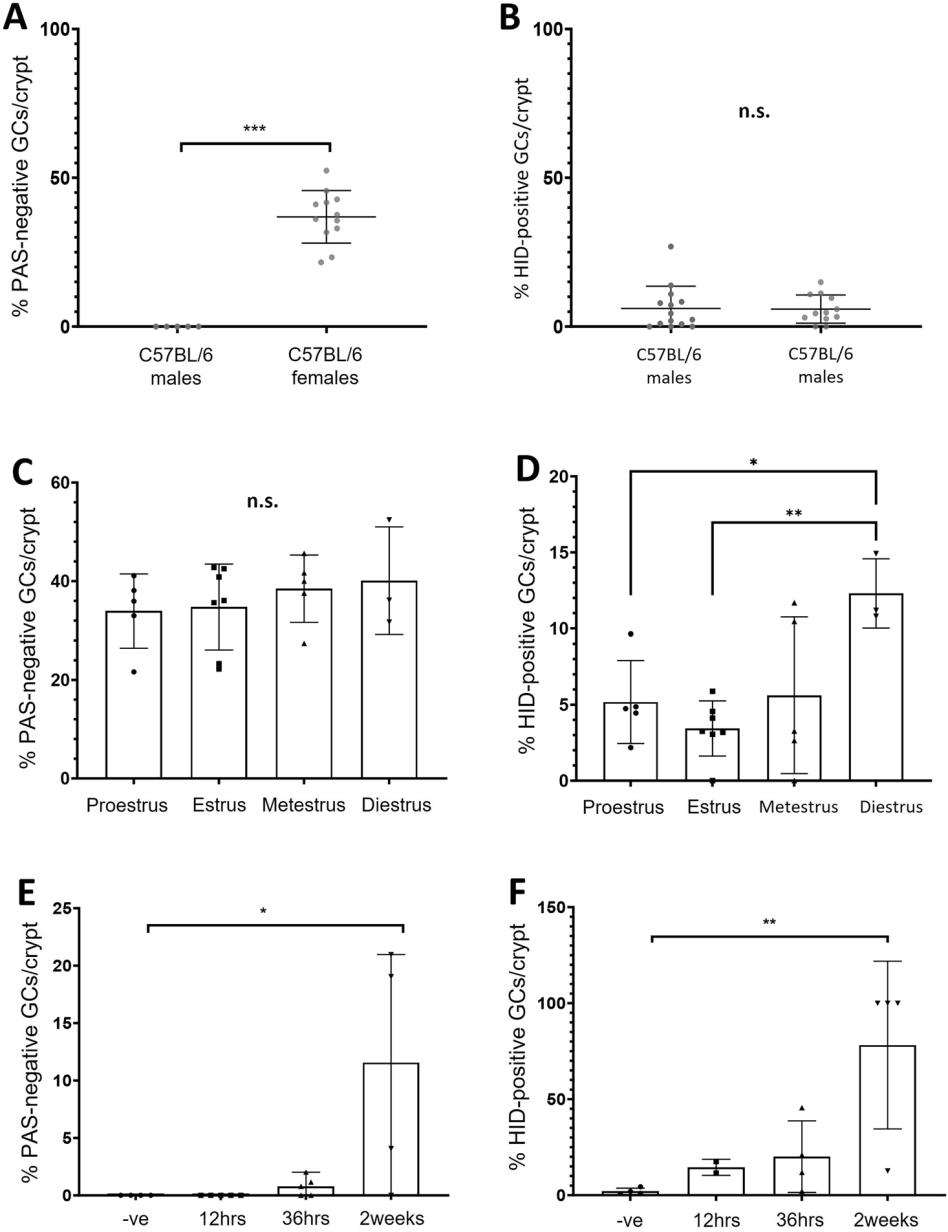
Studies in laboratory animals indicate sex differences in colonic mucin glycosylation are primarily mediated by sialylation, though sulphation is also altered during the estrus cycle in females. The proportion of PAS-negative goblet cells (GCs) per colonic crypt in male and female C57BL/6 mice following AB + PAS staining (**A**). The proportion of HID-positive GCs per colonic crypt in male and female C57BL/6 mice (**B**). Following estrus cycle stage determination, the proportion of PAS-negative GCs (**C**) and HID-positive cells (**D**) was determined via chemical histology in colonic tissue. Finally, male C57BL/6 mice were fed an estrogenic diet of sunflower seeds for 12 h, 36 h or two weeks, and PAS-negative (**E**) and HID-positive (**F**) colonic goblet cells were visualized following AB + PAS or HID staining. Each point represents an individual and is the average value of 10 crypts, n = 3–12. Error bars indicate the median ± SD. *p < 0.05, **p < 0.01, ***p < 0.001, *n.s.* no significant difference. Significance determined via a Mann–Whitney U test (**A,B**) or one-way ANOVA test (**C–F**).

Following the observation that sex associates with the proportion of sialomucin-containing goblet cells in wild mice, this was validated in laboratory-housed C57BL/6 mice. Goblet cells in the proximal colons of naive male and female C57BL/6 mice were examined using AB + PAS and HID staining. Differences between male and female laboratory mice were more pronounced than among wild mice, though variation within both sexes was reduced. Male mice presented with no PAS-negative stained goblet cells, whereas in female mice a median of 36.9% (IQR 10.5) of goblet cells stained PAS-negative (Fig. 2A, Suppl. Fig. 5A,B). The extent of mucin sulphation as assessed by HID staining appeared similar among male (median = 3.4%) and female mice (median = 4.6%) (Fig. 2B). This supports our observation from wild mice that mucin sialylation underpins the differences in PAS-negative goblet cell incidence observed between male and female mice.

Next, we explored the role of systemic sex hormone signalling in relation to the observed sex-dependent differences of the colonic goblet cell staining among wild and laboratory mice. Female C57BL/6 mice were staged via histology of vaginal tissue^18^ (Suppl. Fig. 6), and after staging colonic tissue histology performed to characterise the acidic nature of the mucins within goblet cells. Quantifying the percentage of PAS- negative goblet cells did not reveal any differences between estrus cycles stages (Fig. 2C). However, HID staining revealed a cyclical relationship between the extent of mucin sulphation within colonic goblet cells with respect to the estrus cycle stage of female mice, ranging from a median of 3.3% in the estrus stage, to 11.2% in the diestrus stage (Fig. 2D, Suppl. Fig. 5C,D).

### An estrogenic diet is able to induce changes in the sulphation of goblet cell mucins

In the wild mouse study, sunflower seeds were used to bait traps, resulting in a short (< 12 h) availability of a novel, phytoestrogen-rich diet. Sunflower seeds contain phytoestrogens which can influence sex hormone signalling in mammals^19^. In order to assess the influence of sunflower seeds upon colonic mucin glycosylation, male C57BL/6 were fed on sunflower seeds for a period of 12 h, 36 h or 2 weeks.

No change in the percentage of PAS-negative goblet cells per crypt was seen at 12 h, although the median percentage of HID-positive goblet cells increased from 1.5 to 14.5% at this timepoint. Further changes in mucin glycosylation were apparent by 2 weeks, with male mice displaying populations of PAS-negative goblet cells as high as 20% in some cases (Fig. 2E, Suppl. Fig. 5E,F). Additionally, an increase in mucin sulphation was observed with 100% of goblet cells containing HID-positive mucins in 3 of the 4 mice at the 2-week time point (Fig. 2F, Suppl. Fig. 5G,H).

### Esr1 expression associates with the expression of sialyltransferase enzymes

**Figure 3:**
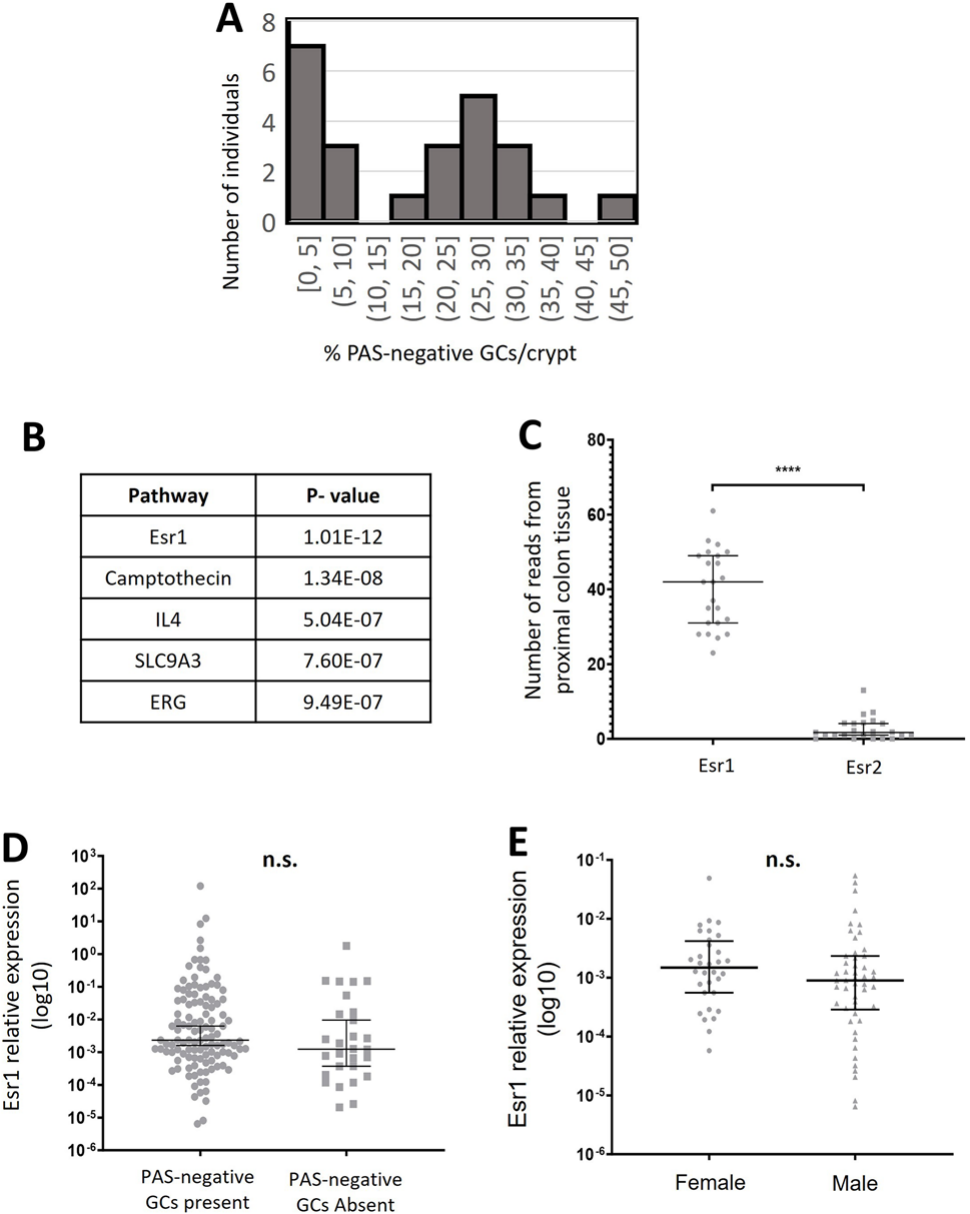
AB + PAS staining in colonic tissue of wild *Mus musculus* is associated with Esr1 receptor expression. RNA sequencing from proximal colon tissue of 24 male mice caught in October 2019 was used for pathway analysis, the percentages of PAS-negative staining of these animals is presented in panel (**A**). The top 1000 most variable genes with respect to AB + PAS staining were used for the prediction of upstream regulators (**B**). The expression of Esr1 and Esr2 determined by RNA sequencing (**C**). The expression of Esr1 in mice with and without a population of PAS negative colonic goblet cells (**D**). The expression of Esr1, determined via qPCR analysis in the 2019 cohort (**E**). n = 24 for sequencing analysis (**B–D**) and n = 114 for qPCR analysis (**E**). ****p < 0.00001, *n.s.* no significant difference, as determined by a Mann–Whitney U test. Correlations determined via a Pearson test.

Transcriptomic analysis was carried out to identify what differential signalling events may be occurring in mice with varying degrees of mucin sialylation in the proximal colon. Twenty-four wild mice were selected for bulk RNA-sequencing analysis of colonic tissue, based upon the results of AB + PAS staining in the colon, which reflects changes in sialic acid and sulphate species within the mucin glycans. Mice were selected with the aim of encompassing a normal distribution of staining ‘profiles’ (Fig. 3A). Sex, month and location were all controlled for within the 24 mice. Using IPA pathway analysis of the top 1000 most variable genes with respect to colour score, an index of AB + PAS staining. Esr1 activation was identified as the strongest predicted regulator of changes in gene expression (Fig. 3B).

RNA sequencing confirmed that Esr1 was expressed in the colonic tissue of mice, while Esr2 was not, as observed in previous publications^19,20^ (Fig. 3C). The expression of other sex-hormone receptors (Esr2, Pgr, Ar) was examined in the RNA sequencing dataset, and were not detected in proximal colonic tissue. Given that Esr1 was linked to PAS-negative goblet cell staining via RNA sequencing analysis, qPCR analysis of proximal colon tissue in the 2019 cohort was conducted to determine if mice possessing greater relative proportions of colonic sialomucins had increased expression of Esr1 (Suppl. Fig. 7). However, levels of Esr1 expression had no association with PAS-negative goblet cell staining (Fig. 3D). Female wild mice also exhibited no significant difference in the relative expression of Esr1 compared to male wild mice (Fig. 3E).

Sialyltransferase enzymes catalyse the transfer of sialic acid from CMP-sialic acid to glycan structures^21^. In the context of mucin O-glycans, common substrates for the sialyltransferase enzymes are *N*-acetylgalactosamine (typically favoured by the St6GalNAc family), and galactose (favoured by the St3Gal family)^22^. The sialyltransferase genes St3gal1, St3gal2, St3gal3, St6galNAc1, St6galNAc2 and St6galNAc4 are all known to be expressed in the mouse colon, therefore the expression of those sialyltransferases was examined via qPCR. Interestingly, all sialyltransferase enzymes examined demonstrated a strong positive association with the expression of Esr1 (Suppl. Fig. 7). To understand the relationship between mucin sulphation and Esr1 expression, sulphotransferase enzymes involved in O-glycan modification were also examined (Gal3St1, Gal3St2, GlcNAc6ST1 and GlcNAc6ST2 (Suppl. Fig. 8A–D). No significant associations were observed.

**Figure 4:**
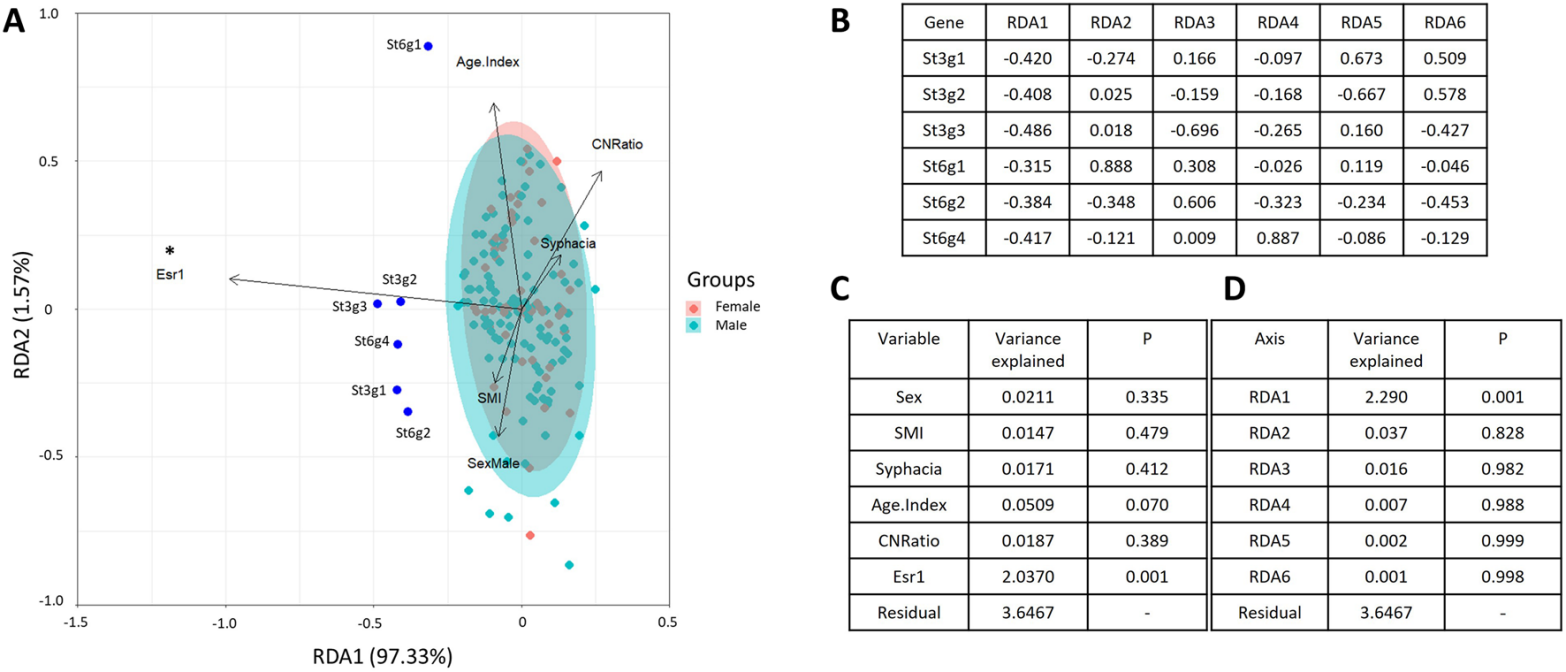
Esr1 expression best describes increases in the expression of sialyltransferase enzymes in the proximal colon of wild *Mus musculus.* The expression of Esr1, as well as the sialyltransferase enzymes: St3gal1, St3g2al, St3gal3, St6galNAc1, St6galNAc2 and St6galNAc4 were examined via qPCR and used in a redundancy analysis (**A**). The loadings of each gene (**B**), variable (**C**) and the contributions of the redundancy axes (**D**). n = 114.

Finally, given the multi-variate environment within which the wild mice live and the many host-intrinsic and extrinsic variables at play, we asked whether certain variables could explain the variation in sialyltransferase enzyme expression via a redundancy analysis. The choice of variables used was informed by the ecological factors found to be most influential on goblet cell staining from general linear models (Suppl. Fig. 3B) as well as Esr 1 expression, diet and age. Esr1 expression was the most highly significant factor (p = 0.001) in describing changes in the expression of sialyltransferases and associated with a general increase in the expression of all the sialyltransferase enzymes quantified (Fig. 4). The age of mice was also approaching significance (p = 0.07) (Fig. 4D), and was associated with the expression of St6galNAc1 (Fig. 4B,C).

## Discussion

This study investigated differences in colonic goblet cells within a population of wild house mice, focusing specifically on the acidic glycan content of mucin-containing goblet cells. By combining histology and ecological information through linear regression models, sex was identified as a strong influencer of mucin glycosylation. Consistently across 2 years, a higher proportion of wild female mice displayed sialomucin-containing goblet cells than wild male mice. Interestingly, under highly controlled conditions in laboratory mice, the sex differences were even more pronounced, with female C57BL/6 mice having significantly more goblet cells containing predominantly acidic mucins than their age matched male equivalents. As in wild mice, the absence of sex differences in HID-staining of colonic goblet cells infers differences in acidity due primarily to sialomucins rather than sulphomucins. Furthermore, our results clearly demonstrate a high heterogeneity in the relative abundance of acidic and neutral glycan content within the colonic mucins of a wild mouse population. This heterogeneity within sexes is not reflected in laboratory C57BL/6 mice. Notably, although this study has focused on the glycosylation of mucins, the presence of other glycoproteins or proteoglycans within goblet cells cannot be ruled out, and may contribute to the histological staining of acidic glycan species observed. However, anti-Muc2 immunofluorescent staining (Suppl. Fig. 4) indicates the major presence of Muc2 within the goblet cells of IoM mice. Moreover, published proteomic data of isolated colonic goblet cells suggests proteoglycans are not produced in abundance.

The more pronounced sex-dependent changes in the acidic glycans in colonic goblet cells observed in laboratory mice compared to wild mice may arise due to the more highly controlled environmental parameters in the laboratory setting, where the effects of sex could be analysed in the absence of other factors such as age and diet. Importantly however, in the multivariate environment in which the wild mice live, significant sex dependent differences in acidic glycan were still apparent. Based on our laboratory experiments and molecular analysis we developed the hypothesis that estrogen signalling modulates mucin sialylation. If estrogen signalling indeed underlies the relationship between sex and goblet cell staining, wild male mice may be encountering more sources of exogenous estrogen (e.g., through diet) than the laboratory housed males resulting in staining characteristics more similar to female mice. Alternatively, the genetic diversity of wild mice may influence the range of staining profiles observed in comparison with inbred mouse strains. We do not know the relatedness of the mice in our study, but previous work on the Isle of May mice has characterised the population as panmictic with a low genetic variability^23,24^. Nonetheless, our study on wild mice demonstrates that whilst the influence of sex may be less pronounced than observed in the laboratory, there is still a quantifiable and reproducible difference in the characteristics of colonic mucin glycans between male and female mice.

Thus far, the effects of estrogen on mucus barriers have primarily focused on reproductive tissues. One study identified positive associations between estrogen and sialomucins, as well as between progesterone and sulphomucins, in the mucus produced by human endocervical cells^25^. The physical properties of the cervical mucus barrier in mammals are known to change during the estrus cycle^26^, and it is possible that cyclical changes in the glycosylation of mucins at reproductive tissues modulates these physical characteristics of the mucus barrier^27^. Furthermore, mucin sialylation in the colon has been shown to be an important mediator of host-commensal homeostasis, with ST6-family enzyme mutations being linked to inflammatory bowel disease in patients and mouse models^5^. An important consideration when interpreting these results is that our study has focused specifically on the proximal colon of mice, which does not necessarily reflect the physiology of other regions of the colon, or the small intestine^28^. Future studies examining sex-mediated differences in other regions of the intestine, or other organs for example the lung, would extend our findings.

Experiments were performed using laboratory models to further explore the association between sex and mucin glycosylation. To investigate the role of systemic sex hormones, the stage of the estrus cycle in female mice was determined. A cyclical increase in colonic mucin sulphation was observed, peaking at the diestrus stage of the cycle. Additionally, male mice fed with an estrogenic diet exhibited an induction in acidic glycosylation of colonic mucins, suggestive of the effects of phytoestrogens on the glycosylation of colonic mucins^29^. Importantly, length of exposure to sunflower seeds in traps on the Isle of May (< 12 h), was not sufficient to drive significant changes in sulphation or sialylation, with significant changes only apparent at the latter two time points of 48 h and 2 weeks. It should also be noted that changes in the mucus barrier arising from altered diet are not necessarily or wholly driven by phytoestrogens. Changes in diet have been reported to have effects upon the glycosylation of mucins, independently of hormonal changes^30^. Furthermore, diet-induced changes in the microbiome also have the potential to influence mucin glycosylation^31,32^, and these aspects were not controlled for in our experiments. Together, these in vivo experiments suggest mucin sialylation is the most important aspect of the sex dependent differences of mucin glycosylation of laboratory animals, and also that mucin sulphation may be associated with systemic sex hormone signalling in male and female mice.

Unbiased molecular analysis of wild animal tissue identified Esr1 as an upstream regulator of alterations in gene expression associated with different histological staining in wild mice. Further molecular analysis was focused upon the expression of sialyltransferase enzymes, revealing a robust association between the expression of Esr1 and sialyltransferase enzymes in wild mice. No significant differences observed in the colonic expression of Esr1, which indicates that putative differences in Esr1 signalling are not mediated by alterations in the transcription of the Esr1 gene product. They may instead be controlled either at the protein level or through differences in Esr1 receptor activation. Future studies would benefit from the examination of either the levels of circulating estrogen in subjects, or the activation of downstream markers of estrogen signalling to provide a more rigorous examination of the hypothesis that acidic mucin glycosylation is increased in response to estrogenic signalling. Furthermore, ecological parameters beyond sex, such as age, may also contribute to the sialylation of mucin glycans.

Alterations in protein glycosylation are implicated in a number of pathological processes. For instance, respiratory viruses such as influenza and coronavirus often bind to sialic acid structures on epithelial cells before invading them^33^. Sex mediated differences in sialic acid expression are proposed to be one mechanism contributing to the increased morbidity and mortality of males during the Covid-19 pandemic^34^. Thus, understanding the drivers of glycosylation in multi-variate environments is an urgent need.

As far as we are aware, we present the first detailed study characterising the colonic mucus barrier, a site naturally important in pathogen defence, in wild house mice. Previous studies have used semi-wild systems where genetically identical laboratory mice have been released into uncontrolled environments^13^, or presented only brief observations^35^. In these studies, no differences were observed in the penetrability or production-rate of colonic mucus in feralised laboratory mice compared to conventionally raised laboratory animals, though increased expression of mucus-barrier associated proteins was observed^13^. Our study illustrates the importance of considering the holistic influence of an animal’s ecological niche, and that elucidating the effects of single parameters on mucus barriers in complex populations benefits from robust ecological study design.

In conclusion, through the use of a wild population of *Mus musculus* and subsequent corroboration in laboratory-housed C57BL/6 mice we show that in a multivariate environment sex is an important determinant of colonic mucin glycosylation. Histological and molecular analysis of the wild mouse population indicated that sex dependent differences in colonic mucin glycosylation among the wild population were at least partly mediated by an association between Esr1 signalling and mucin sialylation. Furthermore, in vivo experiments highlighted variations in colonic mucin sulphation during the estrus, cycle and in response to dietary-estrogen intervention.

## Methods

### Wild mice

Wild *Mus musculus* were live-trapped on the Isle of May (56.18° N 2.55° W). Trapping occurred between November and December of 2018 at 3 trapping grids, and between September and December of 2019 at 2 trapping grids. Trapping grids of 96 Longworth traps were arranged in a 6 × 16 grid, with traps 8–10 m apart. Traps were baited with sunflower seeds and contained Sizzle nesting material (Datesand, CS1A09), as described in Mair et al*.*^17^.

### Laboratory mice

Inbred male C57BL/6 mice (Envigo, UK) aged 6–8 weeks, were housed within the University of Manchester’s Biological Science Facility. Mice were kept in individually ventilated cages under a 12 h light/dark cycle at 22 ± 1 °C and 65% humidity. Unless otherwise stated, animals were fed on a standard chow diet; food and water were provided ad libitum.

### Home office licencing

All experiments were performed on laboratory and wild mice were done in accordance with the UK Animals (Scientific Procedures) Act (1986); the work on laboratory mice was approved by the University of Manchester Local Animal Welfare and Ethical Review Body. The wild mouse study was approved by the University of Nottingham Animal Welfare and Ethical Review Body, and in accordance with the ARRIVE guidelines.

### Alcian blue and PAS (AB + PAS) staining

Tissues were collected using a standardised methodology, with sections for histological analysis taken between 1.5 and 2 cm from the junction of the caecum with the proximal colon. Methacarn-fixed tissues were dehydrated and paraffin-embedded according to standard protocols^36^. Specimens were cut to produce 5 µm sections prior to histological staining. Swiss roll histology tissues were collected by removing faecal pellets and opening murine colons longitudinally. Starting from the distal end, colonic tissue was rolled around a wooden stick, then removed from the stick for fixation.

Sections were dewaxed in citroclear, followed by rehydration down an ethanol gradient (100–50%), to ddH_2_O. Slides were incubated in Alcian blue (3% glacial acetic acid, 1% 8GX Alcian blue at pH2.5) for 5 min, washed, and then incubated in 1% periodic acid for 5 min. Slides were washed again prior to incubation in Schiff’s reagent (Sigma-Aldrich, S5133) for 10 min. After staining with Schiff’s reagent, slides were developed in ddH_2_O for 10 min, then counterstained with Mayer’s Haemotoxilyn (Sigma-Aldrich, MHS32) for 10 s. Finally, slides were washed, dehydrated using an ethanol gradient (50–100%), then cleared in citroclear and coverslipped (using DPX mounting medium (Thermo Fisher Scientific, D/5319/05))^37^.

An index of goblet cell staining, termed colour score was used during the selection of animals for RNA sequencing. Colour score was derived using the following formula:

$$Colour \, score \, = \, \left( {\% \, of \, GCs \, that \, stain \, Light \, blue} \right) - \left( {\% \, of \, GCs \, that \, stain \, magenta} \right).$$

### High-iron diamine (HID) staining

As described for AB + PAS staining, sections were dewaxed and rehydrated. Slides were washed in PBS and incubated in the dark for 18 h with HID solution (0.24% *N,N*,dimethyl-m-phenylenediamine (Sigma 219223), 0.04% *N,N*,dimethyl-p-phenylenediamine (Sigma 193992), 1.68% Iron (III) chloride (sigma 157740)). Slides were then incubated for 5 min in 1% Alcian blue, washed in PBS, dehydrated through an alcohol gradient, cleared with citroclear and coverslips mounted using DPX^38^.

### Determining estrus cycle stage of mice

Vaginal tissue was excised from female mice at the point of culling. Following the Methacarn fixation and AB + PAS staining, vaginal tissue allowed assessment of estrus cycle stage^18^ (Suppl. Fig. 2). Briefly, the presence of mucin containing cells within the epithelium indicated proestrus phase (Suppl. Fig. 2A), shedding of the stratum corneum indicated estrus phase (Suppl. Fig. 2B), leukocyte infiltration indicated metestrus phase (Suppl. Fig. 2C), and a thin stratum corneum without mucin producing cells indicated the diestrus phase (Suppl. Fig. 2D).

### RNA extraction and qPCR

Following excision, colonic tissue was stored in 1 mL of phenol reagent, and immediately frozen with dry ice before being stored at − 80 °C RNA was extracted using standard precipitation protocols using chloroform and isopropanol. Following this cDNA was synthesised from extracted RNA using Tetro cDNA synthesis kit, and primers listed in Table 2 used for qPCR quantification of genes.

**Table 2:** qPCR primer sequences used for analysis of selected gene expression of mouse tissue.

Gene	(5ʹ-) Forward sequence (-3ʹ)	(5ʹ-) Reverse sequence (-3ʹ)
Βeta-actin	GGCTGTATTCCCCTCCATCG	CCAGTTGGTAACAATGCCATGT
Rpl13a	GGGCAGGTTCTGGTATTGGAT	GGCTCGGAAATGGTAGGGG
Esr1	CCCGCCTTCTACAGGTCTAAT	CTTTCTCGTTACTGCTGGACAG
St3Gal1	CCACAACGCTCTGATGGAGG	AACAGTTCCTTGACGGTGTCG
St3Gal2	CTGCTCTTCACCTACTCGCAC	GCTGTAGTCCTGAATAGCCTGG
St3Gal3	AAGCTGGACTCTAAACTGCCT	TGCTGGCTTGGAGAACCTG
St6GalNAc1	ATGGAAGAATGACGCAAGTGC	AACTGACAGAGGTTGTCTCCT
St6GalNAc2	CCTCATGCTGTACTCCTCGG	CTCGTGGGTACGCCATGTT
St6GalNAc4	AGCCTCTTATCCGAGAACTGT	GGCCTGAACCCAGCATCTG

For each primer used an efficiency was calculated as follows:

$${Efficiency \,of\, reaction}= \frac{Primer\, efficiency\, (\%)}{100}+1.$$

A delta Ct value between sample averages and the housekeeping average was calculated. Relative quantity was calculated using the following equation:

$$Relative\, quantity\, \left(RQ\right)=Efficiency\, of \,reaction^{\Delta Ct}.$$

The next step was to determine a geometric mean of the housekeeping gene relative quantity values. This allowed the expression of genes of interest to be described relative to the housekeeping genes using the following formula:

$${Relative \,gene\, expression}= \frac{{RQ}_{gene\, of\, interest}}{Geometric\, mean\, [{RQ}_{housekeeping\, genes}]}.$$

### RNA-sequencing analysis

RNA sequencing was performed and analysed by the Genomic Technologies core facility at the University of Manchester. A notebook containing the scripts used for analysis is provided in https://figshare.manchester.ac.uk/collections/6326849.

### General statistical analyses

All data analyses were carried out using R version 4.3.0. Relevant statistical tests are described in the “Results” section and figure legends.

### General linear modelling

Logistic regression was used for predicting binary outcomes (i.e., presence or absence of PAS-negative goblet cells following AB + PAS staining) from a set of continuous predictor variables. This was performed using the *glm* base R function.

### Redundancy analysis

Redundancy analyses were performed using the *vegan* package in Rstudio. Redundancy analyses was visualised using ordination plots generated with the *ggord* package. Values further than 1.5 × IQR from the median were excluded as outliers. R^2^ is determined using the following formula^39^:

$${R}^{2}= \frac{{SS}_{regression}}{{SS}_{total}}.$$

### Supplementary Information

Supplementary Figures.

## Data Availability

The dataset analysed during this study is available from the Biostudies ArrayExpress repository https://www.ebi.ac.uk/biostudies/arrayexpress/studies/E-MTAB-13517?key=0e70be5c-d0ea-43a6-a3b6-92aa0e8ee5eb (accession number: E-MTAB-13517).
