# Supplementary material for: Sex drives colonic mucin sialylation in wild mice
Source: Sci Rep. 2024 Mar 23;14:6954. doi: 10.1038/s41598-024-57249-x (PMC10960830; doi:10.1038/s41598-024-57249-x)
Supplement: Supplementary file 1 — Supplementary Figures. [file 41598_2024_57249_MOESM1_ESM.pdf]

Supplementary information

Sex drives colonic mucin sialylation in wild mice

Alexander R. Bennett, Iris Mair, Andrew Muir, Hannah Smith, Larisa Logunova, Andrew Wolfenden, Jonathan Fenn, Ann E. Lowe, Janette E. Bradley, Kathryn J. Else and David J. Thornton.

The corresponding authors on this work are:
Alexander R. Bennett - alex.bennett@gu.se
Kathryn J. Else - kathryn.else@manchester.ac.uk
David J. Thornton - dave.thornton@manchester.ac.uk

This file includes 8 supplemental figures.

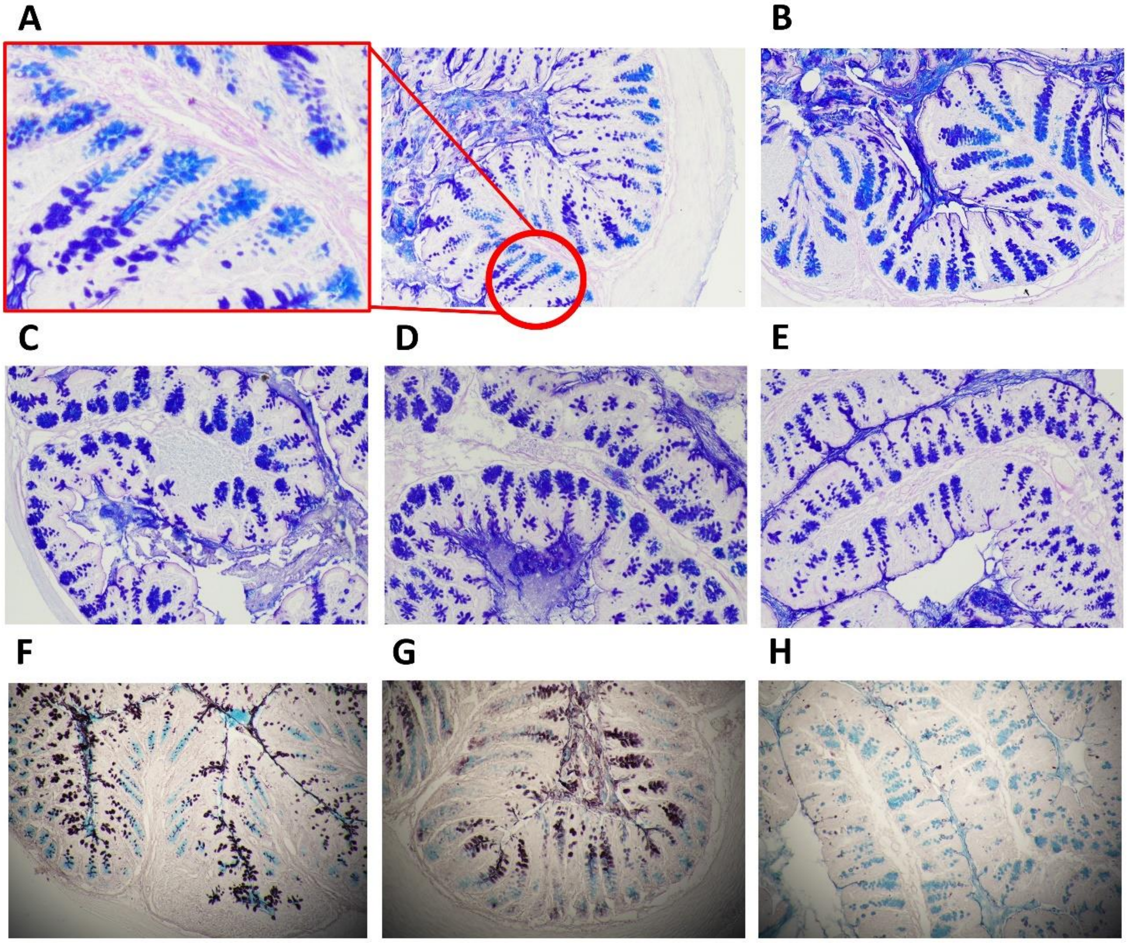

Supplementary figure 1

Representative images of 'Alcian blue + periodic acid Schiff's' (AB+PAS) staining in proximal colon tissue of wild mice with high proportions of PAS-negative staining **(A,B)** and non PAS-negative staining **(C,D,E)**. A high-power image of the region highlighted by the red circle in **A** that highlights the different goblet cell staining profiles. Representative images of the range of 'High-Iron diamine and Alcian blue' (HID) staining observed in the proximal colon **(F,G,H)**.

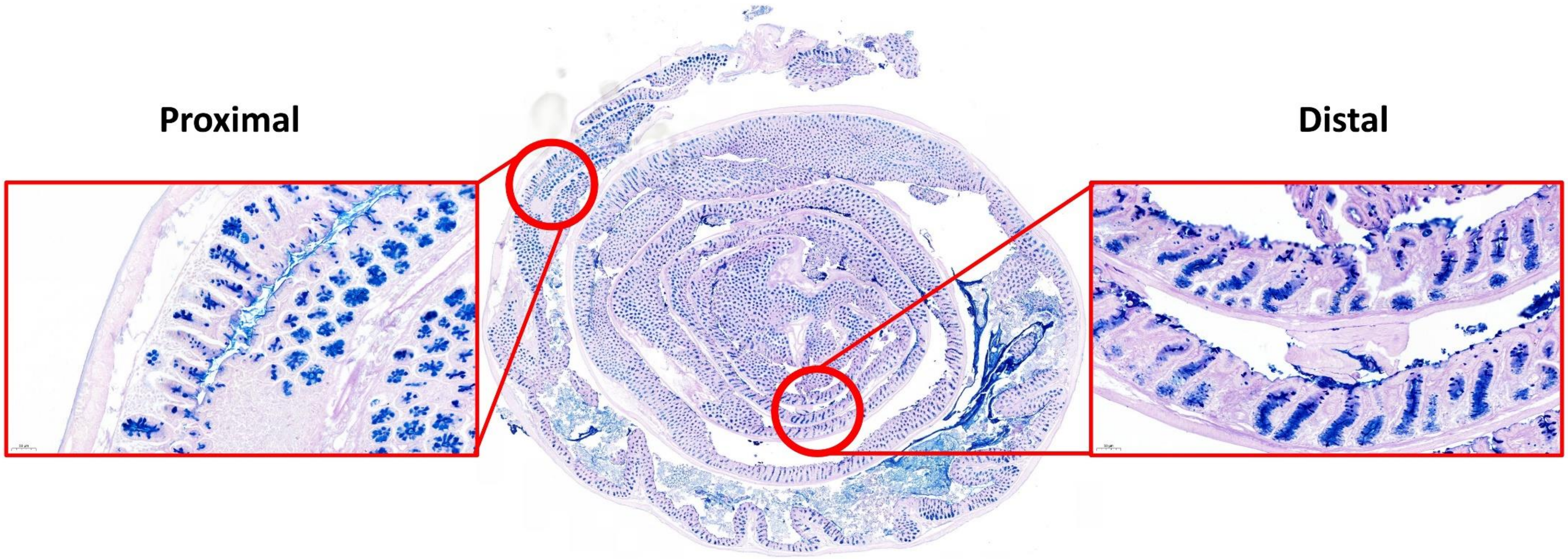

Supplementary figure 2

AB+PAS staining of a colon from an Isle of May mouse outside the study, caught in 2019 (proximal region on the outside of the roll). Panels display the proximal region and the distal region.

A

Factor
2018 / 2019 (Year, catagorical)
Index of an animal's age (MATP1C_CS, numeric)
Male/Female (Sex, categorical)
Scaled mass index (SMI, numeric)
Blood packed cell volume (Blood.PCV, numeric)
Number of syphacia found in the gut (Syphacia, numeric)
Number of Trichuris found in the gut (Trichuris, numeric)
C^{13}/N^{15} Ratio in muscle tissue-representing diet (CN.Ratio, numeric)

B

	Est.	Std error	Adjusted SE	Z value	Pr>Z
**Intercept**	4.655086	2.0082844	2.0138846	2.311	0.0208 *
**SexMale**	-1.1433661	0.4045174	0.4063421	2.814	0.0049 **
**SMI**	-0.1359087	0.1060732	0.1063174	1.278	0.2011
**Syphacia**	-0.0123980	0.0064033	0.0064324	1.924	0.0539 .
**Year19**	0.6094161	0.5857153	0.5868705	1.038	0.2991
**MATP1C_CS**	-0.0507717	0.0953619	0.0955746	0.531	0.5953
**Blood.PCV**	0.0038327	0.0150491	0.0150830	0.254	0.7994
**CN.Ratio**	0.0069660	0.0282873	0.0283781	0.245	0.8061
**Trichuris**	-0.0003064	0.0029739	0.0029856	0.103	0.9183

Supplementary figure 3

The ecological parameters used to construct general linear models of whether an individual in the Isle of May population of wild *Mus musculus* possessed a population of PAS negative goblet cells following AB+PAS staining of the proximal colon **(A)**. The full output of the model **(B).**

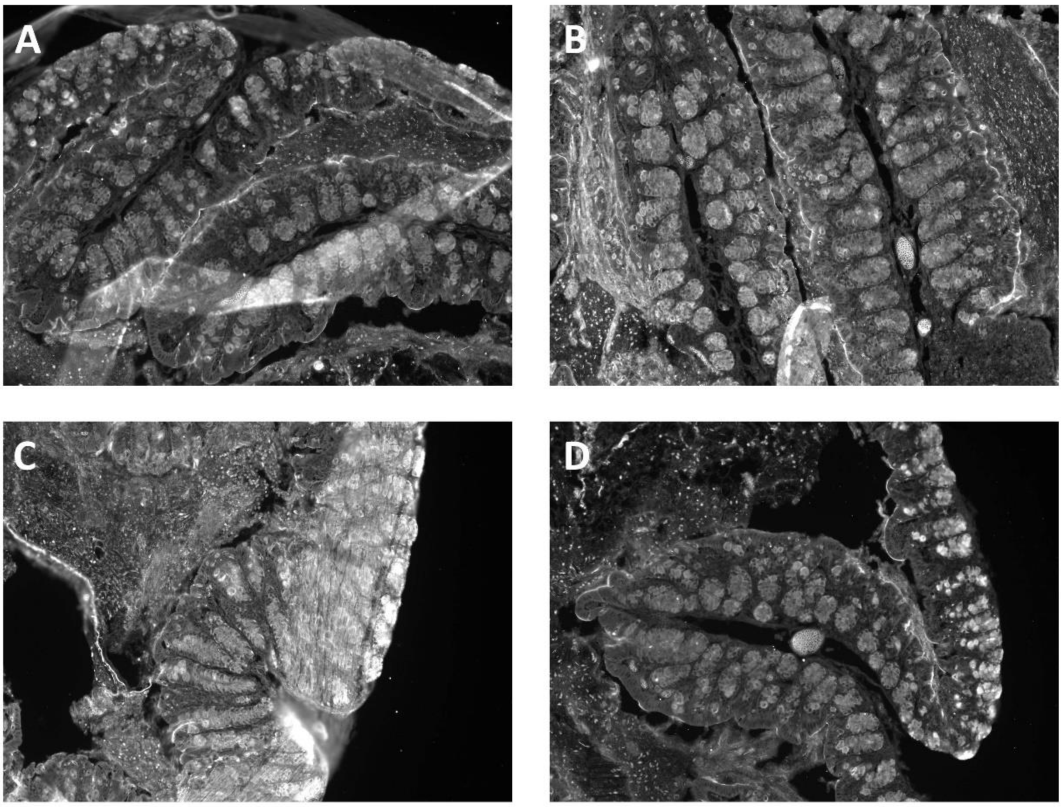

Supplementary figure 4:
Proximal colon tissue sections from four mice caught on the Isle of May (IoM), stained with an anti-Muc2 antibody **(A, B, C, D).**

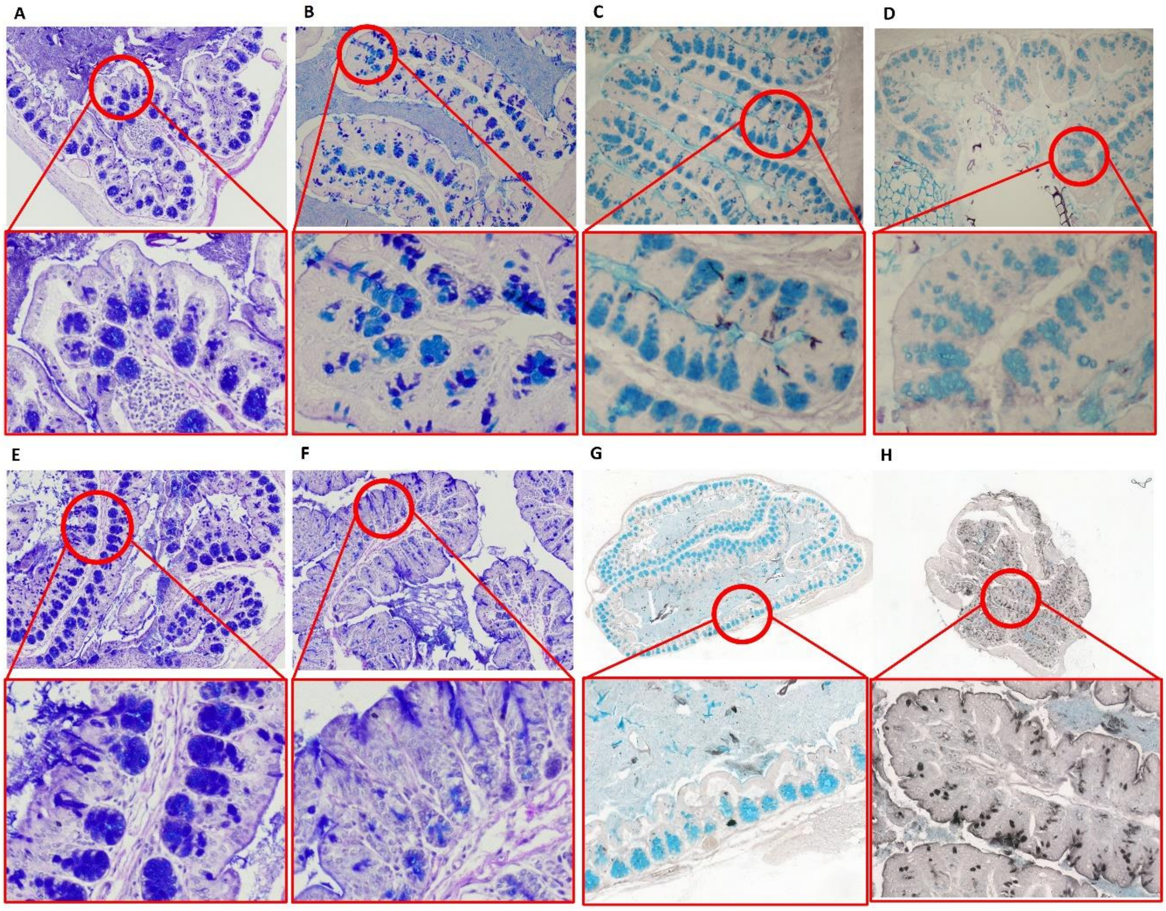

Supplementary figure 5
Representative images of: AB+PAS stained proximal colon tissue from male C57BL/6 mice **(A)** and female C57BL/6 mice **(B),** HID stained proximal colon tissue from C57BL/6 mice in the diestrus phase, with examples of HID+ve cells circled in red **(C)** and the estrus phase **(D).** AB+PAS and HID stained proximal colon tissue of a male C57BL/6 mice on a standard lab diet **(E, G)** and in male C57BL/6 mice fed an estrogenic diet of sunflower seeds for 2 weeks **(F, H).** Lower panels display higher resolution images of the areas encircled in red**.**

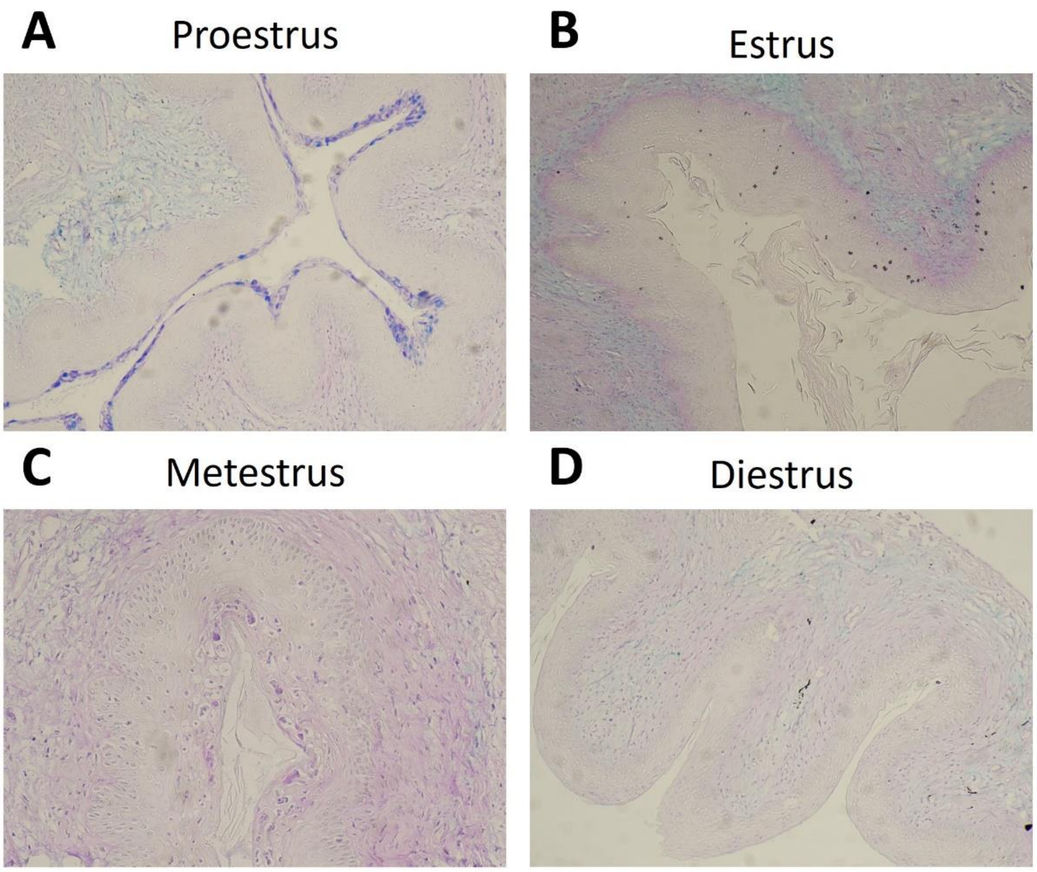

Supplementary figure 6

Representative images of vaginal tissue at each stage of the estrus cycle following AB+PAS staining. Staining was used to determine if mice were in the proestrus **(A)**, estrus **(B)**, metestrus **(C)** or diestrus **(D)** phase of the estrus cycle at the time of cull.

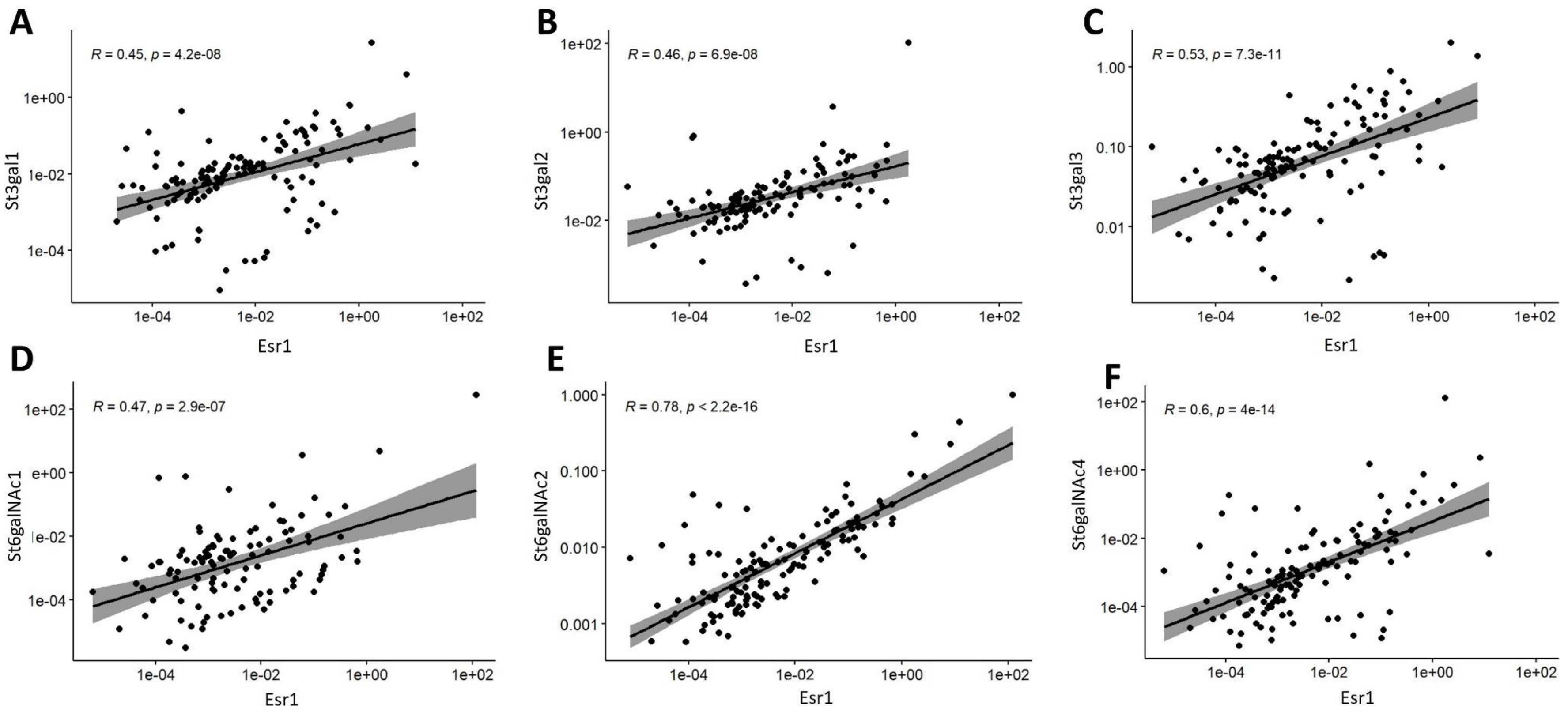

Supplementary figure 7

Correlations between the relative expression of Esr1 and: St3gal1 **(A)**, St3gal2 **(B)**, St3gal3 **(C)**, St6galNAc1 **(D)**, St6galNAc2 **(E)** and St6galNAc4 **(F)** in the 2019 cohort of *Mus musculus* caught on the Isle of May. Correlations performed using a Pearson test. N = 119 – 156.

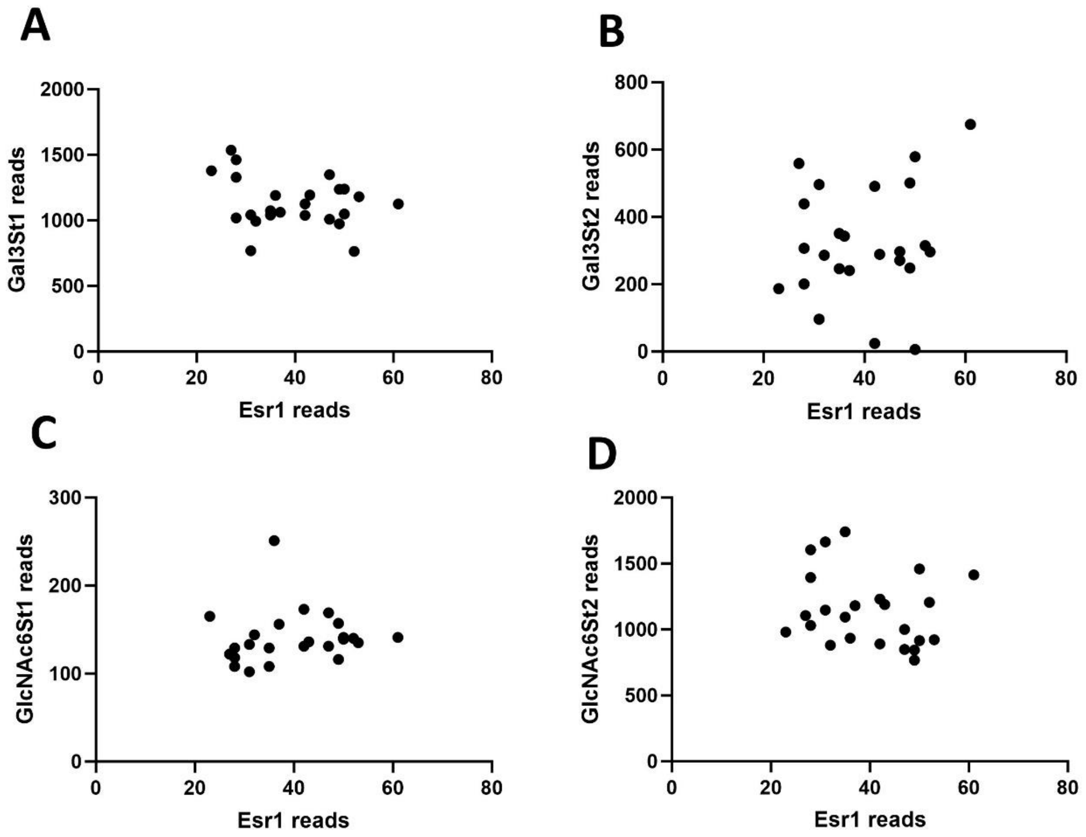

Supplementary figure 8

RNA sequencing reads of Esr1 compared to O-glycan sulphotransferase genes Gal3St1 **(A)**, Gal3St2 **(B),** GalNAc6ST1 **(C)** and GlcNAc6st2 **(D).** N = 24. No correlations were found using a pearson correlation coefficient.
